# High‐Flow Nasal Cannula Versus Conventional Oxygen Therapy in Patients Undergoing Thoracic Surgery: A Randomized Controlled Trial

**DOI:** 10.1111/1759-7714.70251

**Published:** 2026-02-20

**Authors:** Desire T. Maioli, Louise M. Corbellini, Cintia L. Santos, Clovis T. Bevilacqua Filho, Cristiano F. Andrade, Andre P. Schmidt

**Affiliations:** ^1^ Programa de Pós‐graduação em Ciências Pneumológicas, Faculdade de Medicina, Universidade Federal do Rio Grande do Sul (UFRGS) Porto Alegre Rio Grande do Sul Brazil; ^2^ Clinica de Anestesiologia e Tratamento da Dor Bento Gonçalves, Hospital Tacchini Bento Gonçalves Rio Grande do Sul Brazil; ^3^ Serviço de Anestesia e Medicina Perioperatória, Hospital de Clínicas de Porto Alegre (HCPA) Porto Alegre Rio Grande do Sul Brazil; ^4^ Programa de Pós‐graduação em Ciências Cirúrgicas, Faculdade de Medicina, Universidade Federal do Rio Grande do Sul (UFRGS) Porto Alegre Rio Grande do Sul Brazil; ^5^ Departamento de Bioquímica, Instituto de Ciências Básicas da Saúde (ICBS), Universidade Federal do Rio Grande do Sul (UFRGS) Porto Alegre Rio Grande do Sul Brazil; ^6^ Serviço de Anestesia, Santa Casa de Porto Alegre, Universidade Federal de Ciências da Saúde de Porto Alegre (UFCSPA) Porto Alegre Rio Grande do Sul Brazil; ^7^ Serviço de Anestesia, Hospital Nossa Senhora da Conceição Porto Alegre Rio Grande do Sul Brazil; ^8^ Programa de Pós‐Graduação em Anestesiologia, Ciências Cirúrgicas e Medicina Perioperatória, Faculdade de Medicina da Universidade de São Paulo (FMUSP) São Paulo São Paulo Brazil

**Keywords:** high‐flow nasal cannula, oxygen inhalation therapy, postoperative complications, randomized controlled trials, thoracic surgery

## Abstract

**Background and Objective:**

Postoperative pulmonary complications (PPC) are linked to higher morbidity and healthcare costs. High‐flow nasal cannula (HFNC) oxygen therapy may mitigate PPC by enhancing oxygenation and easing respiratory effort. This study assessed HFNC's efficacy versus conventional oxygen therapy in reducing PPC during anesthetic induction and extubation in elective thoracic surgery for lung resection.

**Methods:**

In a single‐center randomized clinical trial, 90 patients undergoing elective thoracic surgery were randomized (1:1) to HFNC or conventional oxygen therapy during induction and extubation. The primary outcome was in‐hospital PPC incidence within 30 days. Secondary outcomes included intubation hypoxemia, 30‐day mortality, and ICU admission. Poisson regression identified PPC predictors.

**Results:**

PPC rates were 20.0% in the HFNC group and 26.7% in controls (relative risk [RR] 0.75, 95% CI 0.35–1.60, *p* = 0.455), with no significant difference. Poisson regression revealed independent predictors: chronic obstructive pulmonary disease, preoperative SpO_2_ ≤ 94%, surgery > 2 h, and left lung ventilation (*p* < 0.05). No differences occurred in intubation hypoxemia (0% both groups), 30‐day mortality (2.22% HFNC vs. 4.44% controls, *p* = 0.553), or ICU admission (13.33% HFNC vs. 17.78% controls, *p* = 0.526). HFNC was well‐tolerated without device issues.

**Conclusion:**

HFNC, applied during intubation and extubation, did not significantly reduce the incidence of PPC or secondary outcomes compared to conventional oxygen therapy in patients undergoing elective thoracic surgery. Further research is needed to explore HFNC's potential in high‐risk populations or with optimized protocols, such as extended application periods or varied flow rates, to enhance perioperative respiratory management.

## Graphical abstract

Short‐term perioperative high‐flow nasal cannula (HFNC) oxygen therapy, applied during anesthetic induction and for 30 min post‐extubation, did not significantly reduce postoperative pulmonary complications compared to conventional oxygen therapy in patients undergoing elective thoracic lung resection surgery.

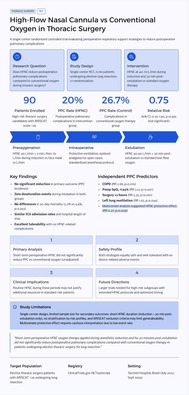

## 1 Introduction

The global surge in surgical procedures, now exceeding 300 million annually, has heightened the demand for advanced perioperative care strategies to better assess risks and minimize postoperative morbidity and mortality [1, 2]. Perioperative complications contribute to a postoperative mortality rate of up to 4.0%, with deaths within 30 days of surgery accounting for 7.7% of global mortality, making postoperative mortality the third leading cause of death worldwide [1, 2, 3]. Beyond the burden of underlying diseases, surgery‐ and anesthesia‐related complications significantly affect patient outcomes, particularly in low‐ and middle‐income countries where access to optimized perioperative care remains limited [2, 4, 5].

Postoperative pulmonary complications (PPC) are among the most frequent and severe adverse events, affecting 1%–23% of surgical patients, depending on the surgery type, patient comorbidities, and diagnostic criteria [6, 7]. PPC encompass a diverse range of conditions, including respiratory infections, respiratory failure, atelectasis, pneumothorax, bronchospasm, pleural effusion, and aspiration pneumonia, which are strongly linked to increased postoperative mortality, extended hospital stays, and higher healthcare costs [8, 9]. Their etiology is multifactorial, driven by patient‐related risk factors, surgical stressors, and anesthetic factors, despite advancements in surgical techniques and anesthetic monitoring [6].

Several evidence‐based strategies have been developed to prevent PPC, including preoperative risk assessment, smoking cessation, pulmonary physiotherapy, protective ventilation techniques, goal‐directed hemodynamic therapy, and early mobilization [10, 11]. A promising intervention in this context is high‐flow nasal cannula (HFNC) oxygen therapy in the perioperative setting. HFNC delivers heated, humidified air‐oxygen mixtures at high flow rates (up to 80 L/min) with adjustable FiO_2_ levels (0.21–1.0), offering physiological benefits such as reduced respiratory effort, enhanced oxygenation, nasopharyngeal dead space washout, mild positive end‐expiratory pressure (PEEP), and improved patient comfort [12, 13, 14].

Despite the widespread use of HFNC in intensive care, evidence for its targeted use in thoracic surgery remains limited [12, 13, 14, 15]. The present study assesses perioperative HFNC versus conventional oxygen in elective thoracic lung resections to reduce in‐hospital PPC, intubation hypoxemia, and short‐term mortality, and explores optimal timing and patient selection.

## 2 Patients and Methods

### 2.1 Study Design

A randomized clinical trial with 1:1 allocation was conducted to compare conventional oxygen therapy and HFNC oxygen therapy. Eligible participants included all adult patients scheduled for elective thoracic surgery at Tacchini Hospital, a regional referral center in southern Brazil, from July 2023 to September 2024. Predefined exclusion criteria ensured sample homogeneity and data relevance. The study received approval from the Tacchini Hospital Ethics Committee, and all participants provided written informed consent after being fully informed about the study's objectives, procedures, and potential risks. The trial was registered on ClinicalTrials.gov (NCT05910788) prior to the first patient enrollment.

### 2.2 Patients

Eligible participants were adults aged ≥ 18 years scheduled for elective thoracic surgery involving lung parenchymal resection, including lobectomy, segmentectomy, wedge resection (therapeutic or diagnostic, including lung biopsy), or metastasectomy, performed via open thoracotomy or video‐assisted thoracoscopic surgery (VATS) at Tacchini Hospital. Patients were excluded if they required emergency surgery, were pregnant, had an ARISCAT score ≤ 26, or declined to participate in the study.

### 2.3 Intraoperative Patient Management

All patients underwent standard monitoring, including continuous electrocardiography, pulse oximetry, noninvasive or invasive blood pressure measurement (as indicated), capnography, neuromuscular monitoring (Train‐of‐Four, TOF), and central temperature assessment. Endotracheal tube size was determined by patient height: women < 1.60 m received a size 35 tube, and those ≥ 1.60 m received a size 37; men < 1.70 m received a size 39, and those ≥ 1.70 m received a size 41. For open surgery, epidural anesthesia was administered at the thoracic T4–T8 level, with ropivacaine 2 mg/mL (6–12 mL), morphine 1 mg, and fentanyl 1 mcg/kg. An epidural catheter was placed before anesthesia induction, and a continuous infusion of ropivacaine (0.1%–0.2%) was initiated immediately after the initial bolus and maintained throughout the intraoperative period. Postoperatively, the infusion was continued up to 48 h if clinically indicated or if intensive care unit (ICU) admission was planned, to provide ongoing analgesia. For laparoscopic procedures, intramuscular methadone (10 mg) was administered at the start of the procedure during patient positioning.

### 2.4 Preoxygenation and Anesthesia Management Protocols

Preoxygenation protocols differed by study group, with both groups positioned with the head elevated to 30° to optimize airway patency and reduce desaturation risk. The control group received preoxygenation using a face mask delivering 10 L/min of oxygen (FiO_2_ 1.0) for 5 min. The intervention group received oxygen via HFNC at 40 L/min (FiO_2_ 1.0) for 5 min, escalating to 70 L/min after induction to maximize oxygen delivery during the apneic phase, prolong safe apnea time, and enhance physiological benefits such as reduced work of breathing and improved oxygenation, based on evidence that higher flows (up to 70 L/min) during induction better match inspiratory demands in respiratory‐compromised patients.

Apnea time was defined as the interval from loss of the capnographic waveform to the first effective ventilation post‐intubation; if manual ventilation was required, it marked the end of the apnea period. This time was measured and recorded by a dedicated research assistant or nurse present in the operating room, using a stopwatch synchronized with the anesthesia monitor. Intubation time was defined as the duration from the start of laryngoscopy to the first effective ventilation, including any repositioning. This time was measured and recorded by a dedicated research assistant or nurse present in the operating room, using a stopwatch synchronized with the anesthesia monitor.

Anesthesia induction was performed as a standardized technique across all patients, regardless of epidural use, using lidocaine 1 mg/kg, propofol 2 mg/kg, fentanyl 2–3 mcg/kg, rocuronium 1.2 mg/kg, and ketamine 0.15 mg/kg. Laryngoscopy began 45–60 s after neuromuscular blockade administration.

Mechanical ventilation was delivered in volume‐controlled mode with tidal volumes of 6–8 mL/kg of predicted body weight, a positive end‐expiratory pressure (PEEP) of 5 to 8 cmH_2_O, an inspiratory pause of 20%, a fraction of inspired oxygen (FiO_2_) of 50%, and a respiratory rate adjusted to maintain normocapnia, consistent with evidence‐based guidelines [16].

During extubation, control group patients underwent conventional extubation and received oxygen via low‐flow nasal cannula or face mask at 2–6 L/min (FiO_2_ 0.3–0.5, titrated to maintain SpO_2_ ≥ 94%). The intervention group received oxygen via HFNC at 40–60 L/min (FiO_2_ 0.3–0.5, titrated similarly) during extubation and for 30 min post‐extubation, followed by a transition to low‐flow nasal oxygen. The extubation protocol followed standard criteria: adequate spontaneous ventilation, reversal of neuromuscular blockade (TOF ratio ≥ 0.9), stable hemodynamics, and core temperature > 36°C. TOF monitoring was used as a criterion for extubation in all patients to ensure full recovery from rocuronium. In cases of oxygenation deterioration requiring airway support, immediate orotracheal reintubation was planned.

### 2.5 Study Interventions and Outcomes

The study comprised two groups: a control group receiving conventional oxygen therapy and an intervention group receiving HFNC oxygen therapy during anesthesia induction and postoperative extubation.

The primary outcome of this study is the incidence of in‐hospital PPC within 30 days among patients undergoing thoracic surgery, comparing those receiving perioperative HFNC therapy with those receiving conventional oxygen therapy. Pulmonary complications include persistent hypoxemia, acute respiratory failure, pneumonia, acute respiratory distress syndrome (ARDS), and pneumothorax. Persistent hypoxemia was defined as peripheral oxygen saturation (SpO_2_) < 90% that persisted for more than 5 min despite supplemental oxygen via nasal cannula, face mask, or higher‐flow devices and required escalation of respiratory support, such as non‐invasive ventilation, reintubation, or increased oxygen flow rate/FiO_2_ [10, 11]. SpO_2_ was continuously monitored using pulse oximetry (Mindray ePM 15 M monitor) from induction through discharge from the postanesthesia recovery room (PACU) or ICU, with events recorded in real time by research assistants, anesthesiologists, or nurses, and escalation determined by clinical judgment.

Secondary outcomes include 30‐day in‐hospital mortality, ICU admission rate, hospital length of stay (LOS), intubation‐related complications (e.g., desaturation during intubation attempts, difficult intubation per Cormack‐Lehane grade), and desaturation events within the first hour after surgical incision. In the present study, desaturation was defined as any episode of SpO_2_ < 90%, regardless of duration, during critical perioperative phases, specifically intubation/extubation and the intraoperative period. Monitoring used continuous pulse oximetry with alarms for SpO_2_ < 90%, from laryngoscopy through the first effective ventilation for intubation desaturation, including laryngoscopy/intubation and the first hour post‐incision.

### 2.6 Statistical Analysis

Sample size was calculated based on an expected incidence of PPC of 30% in the control group and 15% in the HFNC group (an anticipated 50% relative risk [RR] reduction), with 80% power and 0.05 alpha, yielding a minimum of 82 patients (41 per group) [6, 9]. Accounting for a 10% dropout rate, 90 patients were enrolled. Continuous variables were expressed as mean ± standard deviation (SD) or median [interquartile range (IQR)] and compared using Student's *t*‐test or Mann–Whitney *U* test. Categorical variables were presented as absolute values (percentages) and compared using the chi‐square test or Fisher's exact test. Poisson regression with robust variance estimation was used to identify predictors of PPC, with stepwise backward selection (*p* < 0.10 for retention). The primary analysis remains unadjusted; multivariable results are exploratory due to the risk of overfitting. Kaplan–Meier curves and Cox proportional hazards regression assessed time‐to‐event outcomes. Although time to first PPC was not a predefined outcome, we conducted an exploratory Kaplan–Meier analysis to evaluate the temporal distribution of complications. This was performed post hoc to provide additional context on the timing of events, which may inform future studies on the optimal duration of perioperative respiratory support. A *p* value < 0.05 was considered statistically significant. Statistical analyses were conducted using R software, version 4.1.0 (R Foundation for Statistical Computing, Vienna, Austria).

## 3 Results

### 3.1 Patient Enrollment and Group Allocation

**FIGURE 1:**
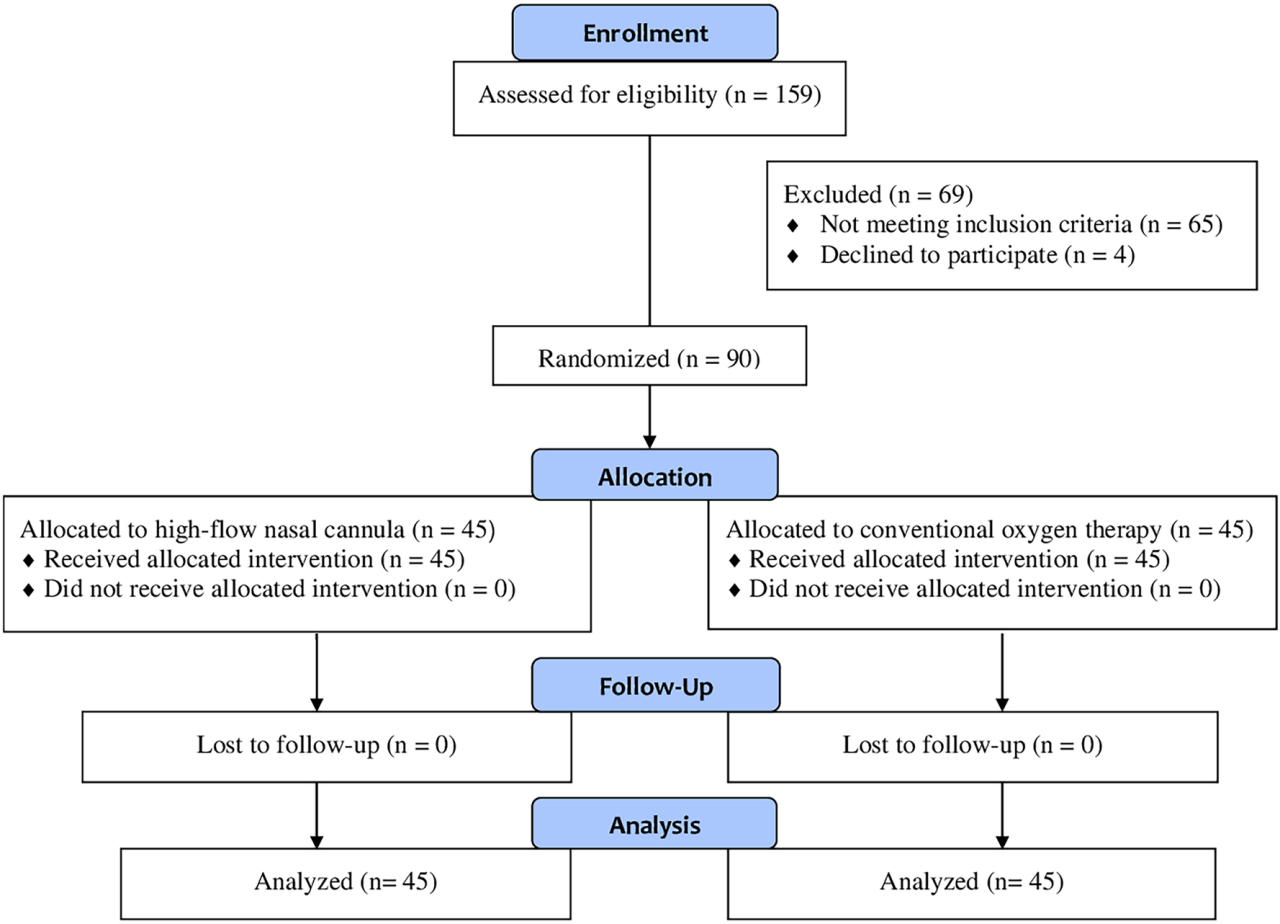
CONSORT flow diagram illustrating the recruitment, allocation, follow‐up, and analysis of patients randomized to receive high‐flow nasal cannula (HFNC) oxygen therapy or conventional oxygen therapy during thoracic surgery.

Of 159 patients assessed for eligibility, 69 (43%) were excluded, primarily due to ARISCAT score ≤ 26 (*n* = 28), emergency surgery (*n* = 19), non‐resection procedures (*n* = 15), or miscellaneous factors (*n* = 3), or patient refusal (*n* = 4). The remaining 90 patients met the inclusion criteria and were randomized in a 1:1 ratio to receive either HFNC therapy or conventional oxygen therapy during anesthetic induction and extubation. The participant flow through the study, including recruitment, allocation, follow‐up, and analysis, is presented in Figure 1 in accordance with CONSORT guidelines.

### 3.2 Baseline Characteristics

Table 1 summarizes the baseline characteristics of the study groups. Demographic variables (age, sex, BMI), surgical risk indices (ASA physical status), types of thoracic procedures (e.g., lobectomy, segmentectomy), and prevalence of comorbidities (e.g., chronic obstructive pulmonary disease—COPD, smoking, cardiovascular diseases) were comparable between the HFNC and conventional oxygen therapy groups, confirming successful randomization. No statistically significant differences were observed in any baseline variable (*p* > 0.05 for all comparisons).

**TABLE 1:** Baseline characteristics of patients undergoing thoracic surgery for lung resection.

	All patients	HFNC	COT	*p*
Gender (*n*—%)				0.830
Male	54 (60.00)	28 (62.22)	26 (57.78)	
Female	36 (40.00)	17 (37.78)	19 (42.22)	
ASA (*n*—%)				1.000
2	50 (55.56)	25 (55.56)	25 (55.56)	
3	40 (44.44)	20 (44.44)	20 (44.44)	
BMI (*n*—%)				0.800
Less than 30	70 (77.78)	34 (75.56)	36 (80.00)	
Greater than 30	20 (22.22)	11 (24.44)	9 (20.00)	
Type of surgery (*n*—%)				0.947
VATS wedge resection	30 (33.33)	15 (33.33)	15 (33.33)	
Open lobectomy	9 (10.00)	5 (11.11)	4 (8.89)	
VATS lobectomy	2 (2.22)	1 (2.22)	1 (2.22)	
VATS metastasectomy	5 (5.56)	3 (6.67)	2 (4.44)	
Open segmentectomy	34 (37.78)	15 (33.33)	19 (42.22)	
VATS segmentectomy	10 (11.11)	6 (13.33)	4 (8.89)	
Age (years—mean ± SD)	63.06 ± 13.90	62.51 ± 14.22	63.60 ± 13.72	0.617
Hypertension (*n*—%)	42 (46.67)	19 (42.22)	23 (51.11)	0.526
CHD (*n*—%)	8 (8.89)	3 (6.67)	5 (11.11)	0.711
Diabetes mellitus (*n*—%)	16 (17.78)	10 (22.22)	6 (13.33)	0.408
COPD (*n*—%)	56 (62.22)	30 (66.67)	26 (57.78)	0.514
Asthma (*n*—%)	6 (6.67)	2 (4.44)	4 (8.89)	0.677
Respiratory Infection < 30 days (*n*—%)	5 (5.56)	1 (2.38)	4 (8.51)	0.362
Smoking (*n*—%)	61 (67.78)	31 (68.89)	30 (66.67)	1.000
OSA (*n*—%)	2 (2.22)	1 (2.22)	1 (2.22)	1.000
Active cancer (*n*—%)	27 (30.00)	12 (26.67)	15 (33.33)	0.646
Anemia (*n*—%)	7 (7.78)	2 (4.44)	5 (11.11)	0.434

*Note:* Comparisons between groups were performed using the *t*‐test or Mann–Whitney *U* test for continuous variables and the chi‐square test or Fisher's exact test for categorical variables. Anemia was defined as preoperative Hb < 10 g/dL. *n* = number of observations; % = percentage.

Abbreviations: ASA = American Society of Anesthesiologists; BMI = body mass index; CHD = coronary heart disease; COPD = chronic obstructive pulmonary disease; COT = conventional oxygen therapy; HFNC = high‐flow nasal cannula oxygen therapy; OSA = obstructive sleep apnea; SD = standard deviation; VATS = video‐assisted thoracoscopic surgery.

### 3.3 Primary and Secondary Outcomes

Analysis of the incidence of PPC showed no statistically significant difference between the HFNC and conventional oxygen therapy groups. In the HFNC group, 20.0% of patients experienced at least one PPC, compared to 26.7% in the control group (RR = 0.75, 95% confidence interval [CI]: 0.35–1.60; *p* = 0.455) (Table 2). Similarly, when considering all pulmonary complications (intraoperative or postoperative), the HFNC group had a complication rate of 24.4% versus 33.3% in the control group (RR = 0.73, 95% CI: 0.38–1.42; *p* = 0.352). Cox regression analysis of time to first complication revealed a hazard ratio (HR) of 0.67 (95% CI: 0.28–1.60) for the HFNC group, but this difference was not statistically significant (*p* = 0.363). These findings indicate that HFNC, applied during anesthesia induction and extubation, did not significantly reduce the incidence or time to the first postoperative complication compared with conventional oxygen therapy.

**TABLE 2:** Incidence of postoperative pulmonary complications within 30 days after surgery in patients undergoing thoracic surgery for lung resection.

	HFNC (*n* = 45)	COT (*n* = 45)	RR (95% CI)	*p*
Any PPC	9 (20)	12 (26.7)	0.75 (0.35–1.60)	0.455
Pneumonia	4 (8.9)	4 (8.9)	1.00 (0.27–3.75)	1.000
Acute respiratory failure	0 (0)	2 (4.4)	—	0.153
Pneumothorax	2 (4.4)	0 (0)	—	0.153
ARDS	0 (0)	1 (2.2)	—	0.315
Persistent hypoxemia	7 (11.1)	7 (13.3)	1.00 (0.38–2.62)	1.000
Postoperative MV	1 (2.2)	4 (8.9)	0.25 (0.03–2.15)	0.167
Any pulmonary complication*	11 (24.4)	15 (33.3)	0.73 (0.38–1.42)	0.352

*Note:* Data are shown as absolute values (percentiles). *p* < 0.05 was considered statistically significant; Pearson's *X* ^2^ test or Fisher's exact test for categorical data (*n* = 45 for the high‐flow nasal cannula [HFNC] group and *n* = 45 for the conventional oxygen therapy [COT] group). Persistent hypoxemia: SpO_2_ < 90% persisting for more than 5 min despite administration of supplemental oxygen (via nasal cannula, face mask, or higher‐flow devices as clinically indicated) and requiring escalation of respiratory support (e.g., non‐invasive ventilation, reintubation, or increase in oxygen flow rate). PPC: postoperative pulmonary complications; Any pulmonary complication* = intraoperative and postoperative pulmonary complications.

Abbreviations: 95% CI = 95% confidence intervals; ARDS = acute respiratory distress syndrome; MV = mechanical ventilation; RR = relative risk.

Secondary outcomes are presented in Table 3. No instances of desaturation during intubation (SpO_2_ < 90%) were observed in either the HFNC or control group. Of the 90 patients, 12 were classified as having difficult intubation, with no significant difference between groups (*p* > 0.05). During hospitalization, 2 patients (4.44%) in the conventional oxygen therapy group and 1 patient (2.22%) in the HFNC group died. However, no statistically significant differences were found between the HFNC and control groups for in‐hospital mortality (*p* = 0.553), ICU admission (*p* = 0.526), or hospital LOS (median 3 days [IQR 2–5] in HFNC vs. 3 days [IQR 2–4] in control, *p* = 0.924) (Table 3).

**TABLE 3:** Incidence of secondary outcomes within 30 days after surgery in patients undergoing thoracic surgery for lung resection.

	HFNC (*n* = 45)	COT (*n* = 45)	RR (95% CI)	*p*
30‐day in‐hospital mortality (*n*)	1 (2.2)	2 (4.4)	0.66 (0.13–3.31)	0.557
ICU admission (*n*)	6 (13.3)	8 (17.8)	0.84 (0.44–1.59)	0.560
LOS (days)	3 (2–5)	3 (2–4)	—	0.924
Hypoxemia during intubation (*n*)	0 (0)	0 (0)	—	1.000
Difficult intubation (*n*)	4 (8.9)	8 (17.8)	0.63 (0.28–1.45)	0.215
Desaturation (*n*)	2 (4.4)	3 (6.7)	1.21 (0.58–2.56)	0,645

*Note:* Secondary outcomes in patients undergoing elective thoracic surgery, comparing high‐flow nasal cannula (HFNC) and conventional oxygen therapy (COT) groups. Outcomes include 30‐day in‐hospital mortality, ICU admission, length of hospital stay (median [interquartile range]), hypoxemia during intubation (SpO_2_ < 90%), and difficult intubation. Desaturation was defined as any episode of SpO_2_ < 90% occurring during laryngoscopy/intubation or within the first hour after surgical incision. Data are presented as absolute values (percentiles) or median (interquartile range [IQR]). *p* values were calculated using chi‐square or Fisher's exact tests for categorical variables and the Mann–Whitney *U* test for length of hospital stay (LOS).

As shown in Table 4, no statistically significant differences were observed between the HFNC and conventional oxygen therapy groups across intraoperative variables, including intubation time, SpO_2_ during intubation, respiratory rate, and durations of single‐lung ventilation, anesthesia, and surgery (*p* > 0.05). These findings indicate that HFNC did not substantially influence intraoperative outcomes. Notably, HFNC was well tolerated, with no reports of patient discomfort or complications, and no device‐related adverse events occurred.

**TABLE 4:** Intraoperative and anesthesia‐related variables in patients undergoing thoracic surgery for lung resection.

	HFNC (*n* = 45)	COT (*n* = 45)	RR (95% CI)	*p*
Intubation time (s)	22 (17–26)	23 (20–32)	—	0.066
Duration of single‐lung ventilation (min)	41.5 (30–63)	45 (30–65)	—	0.878
Surgery duration (h)	60 (47–80)	57 (45–85)	—	0.359
Anesthesia duration (h)	105 (86–130)	95 (80–120)	—	0.318
Double‐lumen tube (*n*—%)	44 (97.8)	41 (91.1)	2.59 (0.44–15.12)	0.361
Epidural analgesia (*n*—%)	25 (55.6)	20 (44.4)	1.25 (0.82–1.90)	0.292

*Note:* Intraoperative and anesthesia‐related variables in patients undergoing elective thoracic surgery, comparing high‐flow nasal cannula (HFNC) and conventional oxygen therapy (COT) groups. Variables include intubation time, apnea time, SpO_2_ during intubation, respiratory rate, surgery duration, anesthesia duration, selective left lung ventilation, and difficult intubation. Continuous variables are presented as median [interquartile range], and categorical variables as number (percentage). *p* values were calculated using the Mann–Whitney *U* test for continuous variables and the chi‐square test for categorical variables.

**FIGURE 2:**
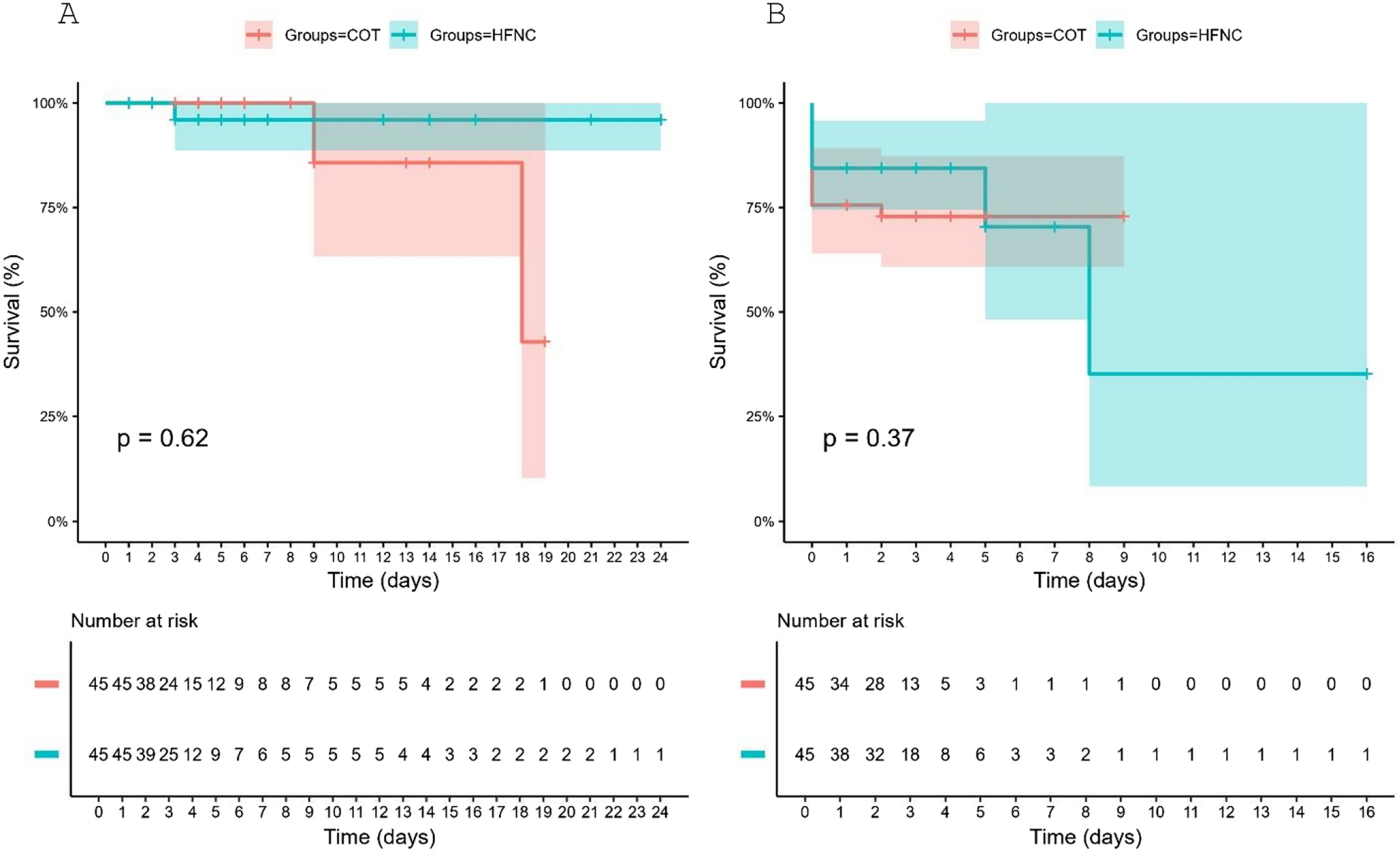
Kaplan–Meier curves for (A) 30‐day in‐hospital mortality and (B) postoperative pulmonary complication (PPC)–free survival in patients undergoing elective thoracic surgery, comparing high‐flow nasal cannula (HFNC) and conventional oxygen therapy (COT) groups. Log‐rank test *p* values are shown for each comparison. Numbers at risk at each time point are provided below the graphs.

The time‐to‐event analysis for 30‐day mortality and PPC, shown in Figure 2 using Kaplan–Meier survival curves, revealed no statistically significant difference between the HFNC and control groups (log‐rank *p* = 0.62). Stepwise backward Poisson regression analysis identified four variables that were significantly associated with the number of PPC: COPD, preoperative peripheral oxygen saturation ≤ 94%, surgery duration exceeding 2 h, and selective left lung ventilation. All variables retained statistical significance in the final models (Table 5). Multivariate analysis also revealed that the use of HFNC during anesthesia induction and extubation was independently associated with a 63% reduction in the RR of PPC (RR = 0.37; 95% CI: 0.18–0.74; *p* = 0.006), suggesting a potential protective effect when applied perioperatively. Notably, these analyses are descriptive given the low event rate.

**TABLE 5:** Predictors of postoperative pulmonary complications in patients undergoing thoracic surgery for lung resection.

	Relative risk (RR)	95% CI	*p*
Use of HNFC	0.37	0.18–0.74	0.006
COPD	2.99	1.37–7.51	0.010
Pre‐operative SpO_2_ ≤ 94%	3.12	1.71–5.78	< 0.001
Surgery duration > 2 h	5.35	2.55–10.94	< 0.001
Selective left‐lung ventilation	1.93	1.03–3.77	0.044

*Note:* Independent predictors of postoperative pulmonary complications (PPC) identified by stepwise backward Poisson regression in patients undergoing elective thoracic surgery for lung resection. Covariates include use of high‐flow nasal cannula (HFNC), chronic obstructive pulmonary disease (COPD), preoperative peripheral oxygen saturation (SpO_2_) ≤ 94%, surgery duration > 2 h, and selective left lung ventilation. Relative risks (RR) with 95% confidence intervals (95% CI) and *p* values are presented for the final model. A *p* value < 0.05 was considered statistically significant.

## 4 Discussion

In this randomized controlled trial, we investigated the efficacy of HFNC oxygen therapy compared to conventional oxygen therapy in reducing PPC in 90 patients undergoing elective thoracic surgery. The primary outcome revealed no significant difference in PPC incidence within 30 days, with 20.0% of HFNC patients and 26.7% of control patients affected. Secondary outcomes, including hypoxemia during intubation, 30‐day mortality, and ICU admission, also showed no significant differences. These findings suggest that short‐term perioperative HFNC does not significantly reduce PPC or improve secondary outcomes compared to conventional oxygen therapy in this patient population. Interestingly, Poisson regression identified COPD, preoperative oxygen saturation ≤ 94%, surgery duration > 2 h, and selective left lung ventilation as independent predictors of PPC in this population.

Although most of the current evidence on HFNC comes from intensive care settings, its application in the perioperative environment shows promise [15, 17]. HFNC oxygen therapy has emerged as a promising alternative to conventional oxygen therapy and NIV for perioperative respiratory management [17, 18, 19, 20]. This modality delivers heated, humidified oxygen at flow rates exceeding 60 L/min through specialized nasal cannulas, offering significant physiological benefits, including enhanced oxygenation, alveolar recruitment, and improved mucociliary clearance [17, 21, 22, 23]. Compared to NIV, HFNC provides greater patient tolerability and requires less intensive monitoring, making it a valuable tool in clinical settings [24, 25, 26]. However, the actual clinical benefits at the surgical setting, optimal timing of use, patient selection, and specific surgical populations for HFNC use still need to be clearly defined.

The physiological benefits of HFNC therapy include nasopharyngeal dead space washout, which reduces hypercapnia, decreases the work of breathing, and delivers heated, humidified gas that lowers metabolic ventilatory demands. Additionally, HFNC generates mild positive end‐expiratory pressure (PEEP), aiding in the prevention of atelectasis [21, 22, 23]. These mechanisms suggest that prophylactic HFNC use could mitigate PPC, particularly in high‐risk patients undergoing thoracic procedures such as thoracotomy with lung resection. However, our findings indicate that HFNC, when applied solely during intubation and up to 30 min after extubation, did not significantly reduce PPC incidence compared with conventional oxygen therapy, underscoring the need to explore optimal application protocols and patient selection criteria.

This study pioneered an innovative approach by employing HFNC therapy from the intraoperative phase, starting at intubation and extending for 30 min post‐extubation. Although infrequently reported in the literature, this strategy appears to enhance respiratory stability during the critical periods of intubation and anesthetic recovery. A previous systematic review reported that preventive HFNC use, compared with conventional oxygen therapy, in patients extubated after lung resection did not significantly improve key clinical outcomes, such as postoperative hypoxemia, reintubation rates, need for escalated respiratory support, postoperative PaCO_2_ levels, hospital LOS, or ICU duration [15]. However, HFNC significantly improved the oxygenation index within the first 12 h post‐extubation in lung resection patients, suggesting benefits for specific respiratory parameters [15]. In contrast, the absence of significant findings for clinically meaningful perioperative outcomes in our study warrants cautious interpretation. Nevertheless, the observed trend toward reduced complications in the HFNC group suggests that early and sustained application of HFNC might confer protective effects in perioperative care, particularly for patients at higher risk of respiratory dysfunction. These findings highlight the need for further research to optimize the timing, duration, and patient selection for HFNC therapy, thereby maximizing its clinical benefits and improving resource utilization.

Multivariate Poisson regression analysis identified several independent predictors of PPC, including chronic obstructive pulmonary disease (COPD; RR 2.99, 95% CI 1.37–7.51, *p* = 0.010), preoperative hypoxemia with SpO_2_ ≤ 94% (RR 3.12, 95% CI 1.71–5.78, *p* < 0.001), surgery duration exceeding 2 h (RR 5.35, 95% CI 2.55–10.94, *p* < 0.001), and selective left lung ventilation (RR 1.93, 95% CI 1.03–3.77, *p* = 0.044; Table 5). These findings are consistent with prior studies that highlight the roles of reduced ventilatory reserve and procedural factors in PPC risk [27]. The strong influence of prolonged surgery duration underscores the need for tailored anesthetic and lung‐protective strategies to mitigate PPC in high‐risk patients.

Although the primary analysis showed no significant difference in PPC incidence between groups (RR 0.75, *p* = 0.455), multivariate Poisson regression suggested a protective effect of HFNC (RR 0.37, 95% CI 0.18–0.74, *p* = 0.006) after adjusting for confounders such as COPD, preoperative SpO_2_ ≤ 94%, surgery duration > 2 h, and selective left lung ventilation. The multivariable Poisson regression suggested a protective association, but this finding should be interpreted cautiously given the low event count and the risk of overfitting. The unadjusted primary outcome showed no significant difference and should guide interpretation. This discrepancy may arise from the model's ability to isolate HFNC's effect in subgroups or from limited sample size reducing power in unadjusted analyses; however, the primary unadjusted result remains the main finding, and the multivariate result should be interpreted cautiously as hypothesis‐generating, warranting confirmation in larger trials.

Regarding secondary outcomes, no significant difference in 30‐day in‐hospital mortality was observed between the HFNC and conventional oxygen therapy groups (2.22% vs. 4.44%; *p* = 0.553), consistent with prior studies that found no mortality benefit of HFNC in specific surgical populations [15, 28]. Similarly, there were no statistically significant differences in hospital LOS or ICU admission rates, aligning with previous research [15, 28, 29, 30]. The limited number of events precludes definitive conclusions, and findings should be interpreted as hypothesis‐generating. These findings may partly stem from the study's limited sample size of 90 patients, which may have constrained the detection of subtle differences. However, they also reflect broader trends in the literature, suggesting that HFNC's impact on these outcomes may be more evident in specific clinical contexts, such as higher‐risk patient groups or settings with extended use of HFNC.

Adequate preoxygenation is a cornerstone of airway management, significantly reducing the risk of desaturation during intubation [31, 32]. In this study, all patients received rigorous preoxygenation protocols, resulting in no episodes of hypoxemia (SpO_2_ < 90%) during intubation in either group. This approach effectively extended safe apnea time, even during difficult or unanticipated intubation, with no recorded desaturation events among the 12 patients classified as having difficult intubations. These findings highlight the critical role of optimized preoxygenation and apneic oxygenation in anesthetic management for thoracic surgeries, such as thoracotomy or VATS, which often involve double‐lumen endotracheal tubes and/or prolonged apnea. The use of HFNC further enhanced this process by enabling hands‐free preoxygenation and a smooth transition to apneic oxygenation, as reported by participating anesthesiologists, potentially improving procedural safety and efficiency during anesthetic induction.

No patients reported discomfort with HFNC therapy, and no device‐related complications occurred during hospitalization, indicating excellent tolerability. A recent randomized study comparing preoxygenation methods found no significant differences in efficacy among standard nasal cannula at 50 L/min with a closed mouth, a well‐fitted face mask, and humidified high‐flow nasal oxygen [33]. Although the standard nasal cannula was less comfortable, its high efficacy underscores the importance of preoxygenation, regardless of the delivery method, particularly when advanced resources like HFNC are unavailable. These findings reinforce the value of accessible preoxygenation strategies in ensuring patient safety during airway management.

Several limitations of the present study should be acknowledged. First, the single‐center design of the trial may limit the external validity of the results, as patient demographics, surgical techniques, and resource availability can vary significantly across institutions and regions. Second, although the sample size of 90 patients was adequate for the primary outcome analysis, it may have been underpowered to detect statistically significant differences in secondary outcomes, such as 30‐day mortality or ICU admission rates, for which only numerical trends were observed. Third, the application of HFNC was limited to the anesthetic induction period and the first 30 min post‐extubation, which may have reduced its potential efficacy compared with more prolonged or continuous administration, an aspect that warrants investigation in future studies. Fourth, the trial did not stratify patients by specific risk profiles, such as COPD severity or other comorbidities, which could influence the effectiveness of HFNC.

Additionally, although preoperative pulmonary function testing and resting peripheral oxygen saturation were routinely assessed, systematic arterial blood gas analysis was not performed in all patients, as many had preserved lung function and no clinical indication for it. This limits our ability to fully evaluate preoperative gas exchange as a potential modifier of HFNC efficacy. Nonetheless, preoperative SpO_2_ on room air was comparable between groups, and preoperative SpO_2_ ≤ 94% was an independent predictor of PPC in multivariate analysis, suggesting that residual imbalances in oxygenation status were unlikely to affect the primary outcome substantially. Furthermore, the ARISCAT score may be suboptimal for thoracic surgery because of the inherently high scores of intrathoracic procedures, and excluding low‐risk patients (ARISCAT score ≤ 26) further limits the applicability of the findings to the broader thoracic surgery population. Finally, the absence of blinding for anesthesiologists and outcome assessors introduces the potential for performance or detection bias, despite randomization having successfully mitigated selection bias. These limitations underscore the need for larger multicenter randomized trials involving more diverse patient populations and optimized HFNC protocols to better define its role in perioperative respiratory management.

In conclusion, this randomized controlled trial demonstrated that short‐term perioperative HFNC oxygen therapy, applied during anesthetic induction and for 30 min post‐extubation, did not significantly reduce the incidence of PPC compared with conventional oxygen therapy in patients undergoing elective thoracic surgery for lung resection. Both strategies were safe, well‐tolerated, and effective, with no significant differences in secondary outcomes, including hypoxemia during intubation, ICU admission, hospital LOS, or 30‐day in‐hospital mortality. These negative findings are clinically relevant, indicating that routine use of HFNC during these specific perioperative periods offers no clear advantage over standard oxygen therapy in patients undergoing thoracic surgeries and may not justify the additional resource requirements in constrained healthcare settings. Larger, multicenter randomized trials with greater statistical power are warranted to confirm these results, particularly in higher‐risk subgroups (such as those with COPD or prolonged surgery), and to explore the potential benefits of optimized HFNC protocols, including extended application durations, higher flow rates, or targeted patient selection, for improving perioperative respiratory outcomes.

## Author Contributions

**Desire T. Maioli:** conceptualization, methodology, software, data curation, investigation, formal analysis, writing of original draft, funding acquisition. **Clovis T. Bevilacqua Filho:** conceptualization, methodology, validation, visualization, writing – review and editing. **Cristiano F. Andrade:** conceptualization, methodology, validation, visualization, supervision, writing – review and editing. **Louise M. Corbellini:** data curation, investigation. **Cintia L. Santos:** data curation, investigation. **Andre P. Schmidt:** conceptualization, methodology, data curation, investigation, validation, formal analysis, supervision, project administration, resources, visualization, writing of original draft, writing – review and editing. All authors read and approved the final manuscript.

## Funding

This work was supported by Tacchini Hospital.

## Conflicts of Interest

The authors declare no conflicts of interest.

## Supporting information


**Data S1:** Checklist.

## Data Availability

The data that support the findings of this study are available from the corresponding author upon reasonable request. De‐identified individual participant data (including data dictionaries) underlying the results reported in this article will be shared for verification or replication of the study's findings. Additional related documents, such as the full study protocol and statistical analysis plan, are also available upon request. Data will become available immediately following publication of the article and will remain accessible indefinitely. Access is granted to researchers who provide a methodologically sound proposal; requests must be made to the corresponding author (apschmidt@hcpa.edu.br) and may require institutional review board (IRB) approval or data use agreement to ensure compliance with Brazilian data protection regulations (LGPD) and ethical standards.
