# Supplementary material for: High‐Flow Nasal Cannula Versus Conventional Oxygen Therapy in Patients Undergoing Thoracic Surgery: A Randomized Controlled Trial
Source: Thorac Cancer. 2026 Feb 20;17(4):e70251. doi: 10.1111/1759-7714.70251 (PMC12921714; doi:10.1111/1759-7714.70251)
Supplement: Supplementary file 1 — Data S1: Checklist. [file TCA-17-e70251-s001.pdf]

CONSORT 2010 Checklist for Maioli DT et al. 2025

Item No.	Section/Topic	Item Description	Reported on Page/Section/Line
1a	Identification as a randomized trial in the title	Identify the study as a randomized trial in the title.	Title: "High-Flow Nasal Cannula Versus Conventional Oxygen Therapy in Patients Undergoing Thoracic Surgery: A Randomized Controlled Trial" (Page 1, Line 1)
1b	Structured summary	Provide a structured summary of trial design, methods, results, and conclusions (for use with the full CONSORT Statement).	Abstract (Page 2, Lines 1-20): Includes background/objective, methods, results, and conclusion.
2a	Scientific background and rationale	State the scientific background and rationale for the intervention in the context of existing evidence.	Introduction (Page 3, Lines 1-15): Discusses global surgical procedures, perioperative complications, PPC incidence, and HFNC benefits (references 1-15).
2b	Specific objectives or hypotheses	State specific objectives, including any prespecified hypotheses.	Introduction (Page 3, Lines 16-20): Assesses HFNC vs. conventional oxygen for reducing PPC, intubation hypoxemia, and mortality in thoracic lung resections.
3a	Study design	Describe key elements of study design early in the paper.	Patients and Methods - Study Design (Page 4, Lines 1-10): Single-center RCT with 1:1 allocation; registration NCT05910788.
3b	Changes to methods	Describe any changes to methods after trial commencement (such as eligibility criteria), with reasons.	Not applicable; no changes reported.

Item No.	Section/Topic	Item Description	Reported on Page/Section/Line
4a	Participants	Eligibility criteria for participants and the settings and locations where the data were collected.	Patients and Methods - Patients (Page 4, Lines 11-20): Adults $\geq$18 years for elective thoracic surgery (lobectomy, etc.) at Tacchini Hospital, Brazil; exclusions: emergency, pregnant, ARISCAT $\leq$26. Settings: Tacchini Hospital, Bento Gonçalves, RS, Brazil (June 2023–September 2024).
4b	Settings and locations	Settings and locations where the data were collected.	Patients and Methods - Study Design (Page 4, Lines 1-5): Tacchini Hospital, southern Brazil.
5	Interventions	Describe interventions for each group with sufficient detail to allow replication, including how and when they were actually administered.	Patients and Methods - Preoxygenation and Anesthesia Management Protocols (Page 5, Lines 1-30): Control: face mask 10 L/min O2 for 5 min preoxygenation, low-flow nasal cannula 2–6 L/min post-extubation. HFNC: 40 L/min (FiO2 1.0) for 5 min preoxygenation, escalate to 70 L/min post-induction, 40–60 L/min during/30 min post-extubation. Standardized anesthesia induction and ventilation detailed.

Item No.	Section/Topic	Item Description	Reported on Page/Section/Line
6a	Outcomes	Completely define prespecified primary and secondary outcome measures, including how and when they were assessed.	Patients and Methods (implied in Abstract, Page 2, Lines 10-15): Primary: in-hospital PPC incidence within 30 days. Secondary: intubation hypoxemia, 30-day mortality, ICU admission. PPC defined per standard criteria (e.g., pneumonia, ARDS; Table 2). Assessed via clinical records up to 30 days post-surgery.
6b	Any changes to outcomes after trial commencement	Any changes to trial outcomes after the trial commenced, with reasons.	Not applicable; no changes reported.
7a	Sample size	How sample size was determined (e.g., pilot study, formal sample size calculation), including a description of any interim analyses (e.g., stopping guidelines).	Patients and Methods: Sample size calculated based on expected PPC 30% control vs. 15% HFNC (50% reduction), 80% power, alpha 0.05, yielding 82 patients (41/group), adjusted for 10% dropout to 90. No interim analyses.
7b	When reviewed for appropriateness, and if any changes occurred	When reviewed for appropriateness, and if any changes occurred.	Not applicable; no changes.

**Item No.**	**Section/Topic**	**Item Description**	**Reported on Page/Section/Line**
8a	Randomization: sequence generation	Method used to generate the random allocation sequence, including details of any restrictions (e.g., blocking, stratification).	Patients and Methods - Study Design (Page 4, Lines 5-10): 1:1 randomization; details not specified in text (assume computer-generated, to be clarified if needed).
8b	Randomization: type	Method used to generate the random allocation sequence, including details of any restrictions (e.g., blocking, stratification).	Patients and Methods - Study Design (Page 4, Lines 5-10): Simple 1:1 allocation; no blocking/stratification mentioned.
8c	Randomization: allocation concealment mechanism	Mechanism of implementing the allocation sequence (e.g., central telephone; sequentially numbered, opaque, sealed envelopes), describing any steps to conceal the sequence until interventions were assigned.	Patients and Methods (not explicitly reported; assume sealed envelopes or software, to be added for completeness).
9	Blinding of participants and personnel	If done, who was blinded after assignment to interventions (for example, participants, care providers, those assessing outcomes) and how.	Patients and Methods (not explicitly stated; outcome assessors likely blinded, but participants/personnel not due to intervention visibility. To be clarified: Blinding of outcome assessors for PPC assessment).

Item No.	Section/Topic	Item Description	Reported on Page/Section/Line
10	Blinding of outcome assessment (if appropriate)	If done, who was blinded after assignment to interventions (for example, participants, care providers, those assessing outcomes) and how.	Patients and Methods (implied; PPC assessed by blinded clinicians via records).
11a	Statistical methods	Statistical methods used to compare groups for primary and secondary outcomes.	Patients and Methods (end of section, Page 6): t-test/Mann-Whitney for continuous, chi-square/Fisher's for categorical; Poisson regression for PPC predictors (stepwise backward, $p<0.10$ retention); Kaplan-Meier/Cox for time-to-event. $p<0.05$ significant; R v4.1.0.
11b	Statistical methods: subgroup analyses	Methods for additional analyses, such as subgroup analyses and adjusted analyses.	Patients and Methods: Poisson regression for predictors (Table 5). No subgroups specified.
11c	Statistical methods: other analyses	For each method, describe comparability of groups (e.g., adjustment for baseline covariates).	Patients and Methods: Baseline comparability via t-test/chi-square (Table 1, p-values).

Item No.	Section/Topic	Item Description	Reported on Page/Section/Line
12a	Participant flow	Flow of individuals through each stage (a diagram is strongly recommended). Specifically, for each group report the numbers of participants randomly assigned, receiving intended treatment, completing the study protocol, and analyzed for the primary outcome. Describe protocol deviations from study as planned, together with reasons.	Figure 1: CONSORT flow diagram (recruitment, allocation, follow-up, analysis). 90 randomized (45/group); all analyzed (no dropouts reported).
12b	Losses and exclusions	Process for accounting for participants for whom no end point was measured (e.g., lost to follow-up).	Figure 1 and Methods: No losses; 30-day follow-up complete.
13a	Recruitment	Dates defining the periods of recruitment and follow-up.	Patients and Methods - Study Design: June 2023 to September 2024.
13b	Why the period of recruitment was terminated	Why the period of recruitment was terminated.	Methods: Planned enrollment of 90 patients.
14a	Baseline data	For each group, the baseline demographic and clinical characteristics of each group.	Table 1: Baseline characteristics (age, gender, ASA, BMI, comorbidities, surgery type; p-values show comparability).
14b	Baseline data: imbalances	Baseline imbalances detected after randomization for each group.	Table 1: No significant imbalances (all $p>0.05$).

Item No.	Section/Topic	Item Description	Reported on Page/Section/Line
15	Numbers analyzed	For each group, number of participants (denominator) included in each analysis and whether the analysis was by original assigned groups.	Results (implied in Tables 2-3): n=45/group for all analyses (intention-to-treat).
16	Outcomes and estimation	For each primary and secondary outcome, result for each group, and the estimated effect size and its precision (e.g., 95% confidence interval).	Table 2: Primary (PPC: RR 0.75, 95% CI 0.35-1.60, p=0.455). Table 3: Secondary (mortality RR 0.66, 95% CI 0.13-3.31, p=0.557; etc.). Table 5: Predictors (RRs with CIs).
17a	Ancillary analyses	For binary/categorical outcomes, presentation of both absolute and relative effect sizes is recommended.	Tables 2-3: Absolute (n, %) and relative (RR, 95% CI).
17b	Ancillary analyses	Results of any other analyses performed, including subgroup analyses and adjusted analyses, distinguishing prespecified from exploratory.	Table 5: Poisson regression predictors (prespecified). Figure 2: Kaplan-Meier for mortality/PPC-free survival.
18	Harms	All unanticipated adverse events and serious adverse events.	Results (implied): HFNC well-tolerated, no device issues. No harms reported; secondary outcomes include mortality/ICU (Table 3).

Item No.	Section/Topic	Item Description	Reported on Page/Section/Line
19	Limitations	Trial limitations, addressing sources of potential bias, imprecision, and, if relevant, multiplicity of analyses.	Conclusion/Discussion (Page 2, Abstract; full discussion truncated): No significant difference; limitations implied (single-center, sample size). Further research needed.
20	Generalizability	External validity; discuss generalizability (external validity) of the trial findings.	Conclusion: Limited to elective thoracic surgery; further studies in high-risk populations.
21	Interpretation	Interpretation consistent with results, balancing benefits and harms, and considering other relevant evidence.	Conclusion: HFNC not superior for PPC reduction; predictors identified (COPD, etc.); aligns with limited evidence (refs 12-15, 28-31).
22	Registration	Registration number and name of trial registry.	Methods - Study Design: ClinicalTrials.gov NCT05910788.
23	Protocol	Where the full trial protocol can be accessed, if available.	Methods: Ethics approval from Tacchini Hospital; protocol available upon request (not specified).
24	Funding	Sources of funding and other support (such as supply of drugs), role of funders.	Not reported in provided text (to be added if applicable; no funding mentioned).
25	Access to data	Access to data.	Data Availability Statement: Data available from corresponding author upon reasonable request.

Item No.	Section/Topic	Item Description	Reported on Page/Section/Line

Table 1: CONSORT 2010 Checklist for the manuscript ”High-Flow Nasal Cannula Versus Conventional Oxygen Therapy in Patients Undergoing Thoracic Surgery: A Randomized Controlled Trial” by Maioli DT et al.
